# **Understanding nutrient imbalances in maize (Zea maysL.) using the diagnosis and recommendation integrated system (DRIS) approach in the Maize belt of Nigeria**

**DOI:** 10.1038/s41598-021-95172-7

**Published:** 2021-08-06

**Authors:** Kamaluddin T. Aliyu, Jeroen Huising, Alpha Y. Kamara, Jibrin M. Jibrin, Ibrahim B. Mohammed, Generose Nziguheba, Adam M. Adam, Bernard Vanlauwe

**Affiliations:** 1 International Institute of Tropical Agriculture, PMB 5320, Ibadan, Oyo Nigeria; 2 Department of Agronomy, Bayero University Kano, Kano, 70001 Nigeria; 3 Department of Soil Science, Bayero University Kano, Kano, 70001 Nigeria; 4 Centre for Dryland Agriculture (CDA), Bayero University Kano, Kano, 70001 Nigeria; 5 International Institute of Tropical Agriculture (IITA), P.O. Box 30772-00100, Nairobi, Kenya

**Keywords:** Plant sciences, Environmental sciences

## Abstract

Low nutrient use efficiency in maize as a result of imbalanced nutrition has been reported to drastically reduce yield. We implemented a nutrient omission experiment to assess the effect of nutrient application on maize yield and nutritional balance. Maize ear leaves were analyzed for nutrients, to identify nutrient balance status using the Diagnostic and Recommendation Integrated System (DRIS) approach. Results indicated that omission of N or P resulted in highly imbalanced DRIS indices respectively, and significantly lower grain yield. A strong inverse relationship between K ear leaf content with DRIS index suggests that K application negatively increases K imbalance in many situations. Imbalances of Mg, Ca and Cu were more associated with higher yielding treatments. A Which-Won-Where result show that nutrient imbalances in the diagnosis were systematically frequent when N was omitted. All the diagnosed nutrients were imbalanced even under the highest yielding NPKZn treatment; indicating further opportunity for yield increase with more balanced nutrition. Balanced nutrition of maize in the maize belt of Nigeria should target application of varying rates of N, P, K, Mg, S and Zn, depending on the soil conditions. But, because of complexities of nutrient interactions during uptake, it is hardly possible to realize a balanced nutrition. However, differentiating the application of antagonistic nutrients into foliar or soil-based methods is recommended for a more balanced maize nutrition.

## Introduction

Genetic improvement of maize (*Zea mays* L.) to tolerate environmental stresses has promoted its cultivation, even into semi-arid and arid parts of Nigeria^1,2^ where growing conditions were less suitable for maize because of high temperature and low rainfall^3^. Consequently, maize production area in Nigeria increased fourfold from 1960 to 2018^4^. Presently maize occupies 17% of the total arable land and 33% of total area under cereals in the country^4^. Total production has increased over eightfold within the same time span. As a result, Nigeria became the second most important maize producer in Africa after South Africa contributing about 13% of the continent’s total production over the last decade^4^.

Unfortunately, like in many other sub-Saharan Africa (SSA) countries, maize productivity per unit area in Nigeria is low at < 2 t ha^−1^ on average, representing 19–21% of the crop’s attainable yield (7–8 t ha^−1^) as reported by Shehu et al.^5^. The low yields are in part attributed to inherently low soil fertility condition^6^, limited use of fertilizers and poor nutrient management^7^. Also, apart from the known deficiencies of N, P and K^8,9^, which are addressed by application of the commonly available fertilizers containing N, P and K^7^, deficiencies of Cu, and Zn^10^, B^5^ and S^11^ are currently reported to constrain maize yields, yet number of studies on micronutrients are just few to support for informed fertilizer policies. Unfortunately, use of NPK fertilizer itself is uniformly prescribed per agroecology, disregarding heterogeneity of soils between fields^12–14^. Continuous adoption of this practice leads to serious nutrient imbalances in the soils, low nutrient use efficiency, low nutrient response and reduced crop performance^15^.

Imbalanced nutrient supply affects plant nutrient uptake and utilization, thus reducing crop yield^15^. Therefore, diagnosing nutrient status of plants may help in designing efficient nutrient management practices for improved crop yield. Nutrient diagnosis using leaf samples has served to evaluate the nutritional status of plants based on chemical analysis of the leaf tissue; which is highly sensitive to soil nutrient status^16^. Maize ear leaf, as one of the sites for greater metabolic activity^17,18^, has been identified as a useful organ for diagnosing maize nutritional imbalances and guide for fertilizer recommendation^19^. The use of maize ear leaf for nutrient diagnosis is based on the established finding that the level of nutrients in the leaf is directly related to nutrient supply and not directly to nutrient concentrations in the soil^20^. However, plant nutrient content is a composite effect of many factors that interact to affect it. Therefore, to effectively interpret results of leaf tissue analysis, knowledge of nutrient interaction during uptake becomes necessary.

For this purpose, researchers developed different methods to assess the nutritional status of plants, e.g., the critical value approach (CVA) and sufficiency range (SRA)^21^. Yet, these methods fail to account for nutrient interactions and magnitude of limitation of each nutrient^22^. In addition to that, SRA is sensitive to the stage of plant development, consequently, implying that diagnosis must be done at the same stage for which the reference sufficiency range was established^23,24^. With the aim to correct the defects in the CVA and SRA, Beaufils^22^ developed the “Diagnosis and Recommendation Integrated Systems” (DRIS). The DRIS is based on the calculation of an index for each nutrient, taking into account its interaction with other nutrients^25^. The main advantage of DRIS is that it considers the nutrient contents in ratio with other single elements (e.g., N/P, P/N, N/K, K/N…), thereby, nullifying the problem of biomass accumulation and sensitivity in changes of tissue nutrient concentration as they age^22,25,26^. The use of DRIS in assessing nutritional imbalance of plants also becomes more recognized due to its ability to rank nutrient limitations for plant demand, and herewith making the achievement of nutritional balance possible. The objectives of this paper were to (i) study the effect of nutrient treatments on maize yield, and (ii) diagnose nutrient imbalance using the Diagnosis and Recommendation Integrated System (DRIS) in the maize belt of northern Nigeria.

## Materials and methods

### Site description

The field experiments were conducted during 2017 rainy season within the maize belt of northern Nigeria. Due to sufficient solar radiation, a well-defined rainy season and low incidences of pests and diseases, the area is considered of highest maize production potential in Nigeria^27,28^. The area comprises of five major agro-ecological zones; namely, Derived savanna (DS), southern Guinea savanna (SGS), northern Guinea savanna (NGS), Mid-altitude (MA), and Sudan savanna (SS)^29^. Dominant soils according to FAO/UNESCO^30^ soil taxonomy are Ferric Luvisols, Acrisols, Cambisols, Nitosols, Leptosols and Lixisols. Rainfall is unimodal and decreases in amount from 1500 mm per annum in the Derived savanna to less than 700 mm in the Sudan savanna, and in duration from seven to four months northward^29^. Mean annual temperatures during the planting season range from 26 to 33 °C, respectively throughout the region^29^.

### Experimental design and treatment structure

A nutrient omission experiment was established in thirty farms selected across the study area, where each site was considered as a replicate. Compared with conventional nutrient omission experimental design where nutrient omission is based on macronutrients; N, P and K (Kihara et al.^49^ and Shehu et al.^5^) so that nutrient omission combinations results in NPK (no nutrient is omitted/all nutrients applied), NP (K omitted), NK (P omitted) and PK (N omitted) and a Control treatment (all nutrients omitted/no nutrient applied), we used a special nutrient omission approach which had not only the N, P and K omission treatments, but also NPK+ treatments in which micronutrients S, B and Zn were subsequently added to NPK, resulting in NPKS, NPKB, and NPKZn treatments accommodated in the omission design. Moreover, we added an NPKSZnB treatment which contained all the nutrients in the design. Together this can also be considered a nutrient omission trial both for macro and micronutrients. The addition of NPKSZnB in the designed allows for the evaluation of the degree of micronutrients limitation, besides evaluating the limitation in macronutrients. The plots were laid out in a randomized complete block design; with 3 blocks each consisting 3 plots. Each plot consisted of 6 rows (spaced at 0.75 m apart) of 5 m length to give a gross plot area of 22.5 m^2^. The plots were separated by an unplanted row or 1 m when row spacing was not applicable. The nutrients N, P and K application rates were 140 kg N ha^−1^, 50 kg P_2_O_5_ ha^−1^ and 50 kg K_2_O ha^−1^ respectively. Nitrogen (N) was applied as urea (manufactured by Notore Chemical Industries Plc, Nigeria) in three equal splits, i.e., at planting (basal), at 21 and 42 days after emergence (DAE), while full doses of the other nutrients were applied during planting. P was applied from Triple super phosphate (TSP-manufactured by Elixir Garden Supplies), while K was applied using muriate of potash (MOP produced by Gold Prime Fertilizer Company, Nigeria). Elemental S (locally sourced as pyrite) and Zn (using ZnSO_4_ manufactured by Park scientific limited, UK) were applied at the rate 10 kg ha^−1^ while B was applied at 5 kg ha^−1^ using Borax (U.S. Borax Inc. USA).

### Maize variety, sowing and harvesting

SAMMAZ 15; the most commonly grown maize variety in the study area was used in the experiment. The variety was developed by International Institute of Tropical Agriculture (IITA) and released in 2008 by Institute for Agricultural Research (IAR) in Nigeria. The variety is open pollinated, *Striga*, streak virus and drought tolerant/resistant, with 105–110 maturity days. Sowing was done within the best window for planting maize (June and July) of the area. Two seeds per hole sown at 0.25 m intra-row and 0.75 m inter-row spacing were thinned to one plant per hill between 10 to 14 days after sowing to give an average recommended plant population of 53,333 plants ha^−1^. Plants within a net plot area (two innermost rows minus 1 m from both ends of each row) of 2.25 m^2^ were harvested at full maturity. The harvested plants were separated into stems and cobs, and dried. The dried cobs were shelled and the grains weighed for yield determination. The grain yield was expressed in kg ha^−1^ after weighing the shelled grains and adjusting the moisture to 15% using grain moisture tester as reported by Kamara et al.^2^.

### Soil and Ear leaf sampling and analysis

Three soil samples from each site at 0-20 cm sampling depth were collected using soil auger prior to trial establishment in a ‘V-shape sampling approach’. The samples were then placed in a basin, thoroughly mixed and a composite sample obtained. The composite samples were air dried before chemical analyses at the analytical service laboratory of the International Institute of Tropical Agriculture (IITA) Ibadan, Nigeria. Soil pH water in water (S/W ratio of 1:1) was measured using a glass electrode pH meter and the particle size distribution following hydrometer method. Total soil organic carbon (SOC tot) was measured using a modified Walkley-Black chromic acid wet chemical oxidation and spectrophotometric method. Total nitrogen (N tot) was determined using a micro-Kjeldahl digestion method^31^. Available Phosphorus (P avail.) and Sulphur (S avail.), exchangeable cations (K, Ca, Mg and Na) and micronutrients (Zn, Fe, Mn and B) were determined using Mehlich-3 extraction procedure^32^ and reading with inductively coupled plasma optical emission spectroscopy (ICP-OEC, Optima 800, Winlab 5.5, PerkinElmer Inc., Waltham, MA, USA). Exchangeable acidity (H + Al) was determined by extracting soil with 1N KCl and titration of the supernatant with 0.5M NaOH. Effective cation exchange capacity (ECEC) was calculated as the sum of exchangeable cations (K, Ca, Mg and Na) and exchangeable acidity (H + Al)^33^. For the leaf sampling, ten maize ear leaf samples were randomly collected from the second and fifth rows immediately at the beginning of silk stage (female flower initiation stage). The samples were washed with distilled water and allowed to air dry. The dried samples were then ground with agate pestle and mortar and analyzed for nutrient contents. Nitrogen was analyzed by digesting the samples in hot sulphuric acid solution in the presence of Se as catalyst, followed by colorimetric N analysis using autoanalyzer (Technicon AAII, SEAL Analytical Inc.) following indophenol blue method. For the determination of available Sulphur, ball-milled samples were digested with nitric acid (HNO_3_) and the nutrient contents in the digest were determined in Inductively Coupled Plasma Optical Emission Spectrometry (ICP-OES Optima 3300 DV, Perkin Elmer, Norwalk, USA). Available Phosphorus, cations (K, Ca and Mg) and micronutrients (Zn, Fe, Cu, Mn and B) were analyzed by first dry-ashing the samples for 4 h at 550 °C and then prepared and read on ICP-OES Optima 800, Winlab 5.5 (manufactured by PerkinElmer Inc.,Waltham,MA, USA). All the methods used in this study for plant sampling are in compliance with the IUCN^34^ policy on Research Involving Species at Risk of Extinction, and the Convention on the Trade in Endangered Species of Wild Fauna and Flora.

### DRIS analysis

For establishing the DRIS norms, the data was divided into two sub-populations i.e., a low (non-reference) and high yielding (reference) sub-populations. This was done by first sorting the plots according to yield in decreasing order and then portioning the data into the two sub-populations. The high yielding population comprised plots having grain yield higher than the Mean + 0.5* standard deviation; the mean and the standard deviation were calculated from the entire dataset. Thus, plots with yields ≥ 3766 kg ha^−1^ were considered high yielding, and they constituted 31% of the entire dataset. DRIS norms and coefficients of variations (CVs) were derived according to the procedure by Walworth and Sumner^25^. Mean value for each nutrient pair (example, A/B), their corresponding CV, and variance (σ^2^) were then calculated separately for the two sub-populations. The mean value of each dual nutrient ratio in the high-yielding population were used as DRIS norms^25^. To select the dual nutrient ratio order (A/B or B/A) for calculating the DRIS indices (Eqs. 1 and 2, or 3 and 4), the variance of the low yielding sub-population was divided by that of the high yielding sub-population. It is theoretically expected that the data of the low yielding population would be more unbalanced and therefore should have larger variance than the high yielding one. The dual nutrient ratio with higher variance ratio value were therefore selected and used in the diagnosis. The DRIS indices were calculated based on ratios of each nutrient relative to all other nutrients using the equations below by Walworth and Sumner^35^.

The DRIS index is the mean deviation of the dual nutrient ratios of a given nutrient from their respective norm values. The nutrients were ordered based on their DRIS index value, to determine the order of the degree of limitation to produce the nutrient limitation diagnosis. A DRIS index value for given nutrient close to zero (“0”) indicates nutritional balance for that given nutrient relative to other nutrients in the diagnosis. A more negative index value for a given nutrient indicates lower amount or shortage relative to other nutrients. Alternatively, higher positive index value of a nutrient indicates excess presence of that nutrient relative to others^36^.

If we consider hypothetical nutrients A, B through N, then:

1 $$A\;index = { }\frac{{{\text{f}}\left( {\frac{{\text{A}}}{{\text{B}}}} \right) + {\text{f}}\left( {\frac{{\text{A}}}{{\text{C}}}} \right) + {\text{f }}\left( {\frac{{\text{A}}}{{\text{D}}}} \right) + \cdots + {\text{f}}\left( {\frac{{\text{A}}}{{\text{N}}}} \right)}}{{\text{n}}}$$

2 $$B\;index = { }\frac{{ - {\text{ f}}\left( {\frac{{\text{A}}}{{\text{B}}}} \right) + {\text{f}}\left( {\frac{{\text{B}}}{{\text{C}}}} \right) + {\text{f }}\left( {\frac{{\text{B}}}{{\text{D}}}} \right) + \cdots + {\text{f}}\left( {\frac{{\text{B}}}{{\text{N}}}} \right)}}{{\text{n}}}$$

For, if A/B ≥ a/b;

3 $${\text{f}}\left( {\frac{{\text{A}}}{{\text{B}}}} \right) = \left[ {\frac{{\left( {\frac{{\text{A}}}{{\text{B}}}} \right)}}{{\left( {\frac{{\text{a}}}{{\text{b}}}} \right)}} - 1} \right]{ }{ \times }\frac{1000}{{{\text{CV}}}}$$

or, if A/B < a/b;

4 $${\text{f}}\left( {\frac{{\text{A}}}{{\text{B}}}} \right) = \left[ {1 - { }\frac{{\left( {\frac{{\text{a}}}{{\text{b}}}} \right)}}{{\left( {\frac{{\text{A}}}{{\text{B}}}} \right)}}} \right] { \times }\frac{1000}{{{\text{CV}}}}$$

where a/b is the DRIS norm for the ratio of nutrients A and B, and CV is the coefficient of variation associated with that norm expressed as percentage. A/B denotes the ratio of average concentration of the ten ear leaves collected per plot for nutrients A and B, n is the number of nutrients considered, and* f* (A/B) is a function of nutrients A and B ratio. The 1000 multiplier in Eqs. 3 and 4 comprises of a factor 10 to give the resultant indices a convenient magnitude and a factor 100 to express the CV as fraction rather than as percentage.

### Statistical analyses

Descriptive statistics was carried out to study the distribution of the soil, yield and ear leaf data using mean, range and coefficient of variation (CV). Relationship between respective nutrient ear leaf content and DRIS index was studied using a regression analysis, with nutrient ear leaf content as the independent variable and the DRIS index as the dependent variable. Simple scatter plot of the DRIS indices was plotted to study the relationships among the nutrient DRIS indices. Comparison GGE biplot was drawn with sectors to demarcate most responsible nutrient treatment which influenced nutrient DRIS indices based on mean and stability. All statistical analyses were done in GenStat 17th edition statistical package (VSN International).

## Results

### Physico-chemical soil properties at the experimental sites

Soil properties across the experimental sites showed wide variation, except for pH which has a low coefficient of variation (CV) of 7.90% (Table 1). Average pH value (6.63) indicated that the soils can be generally regarded as slightly acidic. Total soil organic carbon (SOC _tot_) was very low, and this condition seems to be moderately consistent across the sites. Average total nitrogen (N_tot_) (0.65 g/kg) and available phosphorus (P_avail._) (2.40 mg/kg) are rated ‘very low’ according to Nigerian National Special Programme on Food Security NSPFS^37^ and Esu^38^ fertility classification of Nigerian Savanna soils. According to the same fertility rating, average K (0.79 cmol_c_ kg^−1^) is considered optimum to high, Mg (1.25 cmol_c_ kg^−1^) is considered ‘low’ to ‘moderate’, and Ca (3.67 cmol _c_ kg^−1^) Zn (5.02 mg kg^−1^), Fe (118.8 mg kg^−1^), and Mn (151.2 mg kg^−1^) all fall under the ‘optimum’ sufficiency range. Boron (0.09 mg kg^−1^ on average) is rated as ‘very low’. However, the CVs of Mg (66%), B (103%), and Zn (69%) were the highest among the soil properties and further indicate high variation across the sites. The sand percentage was on average 54.70% and did not vary too much, with the texture classes predominantly sandy clay loam and sandy loams according to USDA^39^ textural soil classification.

**Table 1:** Selected physico-chemical properties of top soil (20 cm) layer in the experimental fields.

Soil property	Average	Minimum	Maximum	CV	Soil property	Average	Minimum	Maximum	CV
pH _water_	6.63	5.50	7.60	7.9	S _avail._ (mg kg^−1^)	9.80	3.84	48.68	41.7
SOC _tot_ (g kg^−1^)	6.06	2.30	11.50	31.8	B (mg kg^−1^)	0.09	0.00	0.77	103.1
N _tot_ (g kg^−1^)	0.65	0.21	1.15	34.6	Zn (mg kg^−1^)	5.02	0.07	14.00	68.9
P _avail_. (mg kg^−1^)	2.40	1.03	7.36	51.8	Cu (mg kg^−1^)	2.29	1.47	3.94	34.5
Ca (cmol _c_ kg^−1^)	3.67	0.87	9.17	54.5	Mn (mg kg^−1^)	151.2	14.52	333.4	51.9
Mg (cmol _c_ kg^−1^)	1.25	0.29	4.46	66.1	Fe (mg kg^−1^)	118.8	77.78	182.9	25.7
K (cmol _c_ kg^−1^)	0.79	0.37	1.42	34.7	Sand (g kg^−1^)	540.7	35.0	81.0	21.9
Na (cmol _c_ kg^−1^)	0.10	0.01	0.12	20.3	Silt (g kg^−1^)	210.4	9.0	39.0	35.5
ECEC (cmol _c_ kg^−1^)	5.81	1.64	14.58	47.2	Clay (g kg^−1^)	230.6	10.0	46.0	38.2

### Maize grain yield

Maize grain yield ranged from 101.00 to 7450 kg ha^−1^ across the nutrient treatments (Tables 2 and 3). Except under NPKB plots, average grain yield of the NPK and NPK + treatment plots were above 3,000 kg ha^−1^. The NPKZn treatment increased grain yield by 28% compared to NPK. Omission of either N or K (in PK and NP treatment respectively) both reduced yield by 59.00%, and that of P (NK treatment) reduced yield by 56.00% compared to that of the NPK treatment. On average, yield for the Control plot was below 1500 kg ha^−1^. The highest coefficient of variation (66.00% and 65.00%) of yield under Control and NP, respectively indicated that the experimental fields were highly variable in terms of fertility status.

**Table 2:** Range of maize grain yield and ear leaf concentrations of macronutrients and micronutrients of fertilizer treatments.

Treatment		Yield (kg ha^−1^)	Macronutrients (%)						Micronutrients (mg kg^−1^)				
			N	P	K	Ca	Mg	S	Cu	Mn	Zn	B	Fe
Control	Mean	1445	1.90	0.16	1.03	0.78	0.30	0.63	5.31	111.3	17.7	2.64	39.7
	Min	101	1.31	0.04	0.02	0.06	0.01	0.05	2.90	61.23	12.5	0.33	25.4
	Max	3471	2.61	0.27	1.56	1.03	0.46	1.12	11.25	183.8	27.8	4.09	65.9
	CV (%)	66	17	42	39	29	40	53	42	34	25	39	26
NK	Mean	1745	2.57	0.20	1.33	0.86	0.35	0.61	5.28	152.6	24.0	2.72	38.6
	Min	407	2.24	0.07	0.66	0.68	0.17	0.21	3.43	105.0	9.8	1.58	25.3
	Max	2697	3.14	0.28	2.52	1.18	0.71	1.03	8.82	202.1	37.1	4.01	55.7
	CV (%)	43	11	31	33	15	49	38	24	21	39	30	24
NP	Mean	1612	2.69	0.23	1.03	0.96	0.39	0.62	5.78	153.4	23.4	2.94	40.5
	Min	1177	2.17	0.10	0.46	0.68	0.23	0.04	2.90	90.0	8.0	1.14	22.8
	Max	3465	3.45	0.33	1.91	1.57	0.58	1.25	9.42	225.4	45.5	4.29	68.3
	CV (%)	65	12	24	33	28	29	62	34	23	38	27	27
PK	Mean	1621	1.77	0.21	1.04	0.98	0.43	0.55	4.27	109.6	14.7	2.74	38.9
	Min	982	1.28	0.12	0.39	0.62	0.19	0.04	2.25	49.3	8.4	0.96	22.8
	Max	2602	2.39	0.29	1.64	1.65	1.46	1.26	7.59	188.0	27.3	3.75	65.9
	CV (%)	28	17	27	33	25	74	55	33	38	39	25	30

**Table 3:** Range of maize grain yield and ear leaf concentrations of macronutrients and micronutrients of fertilizer treatments.

Treatment		Yield (kg ha^−1^)Yield (kg ha^−1^)	Macronutrients (%)						Micronutrients (mg kg^−1^)				
			N	P	K	Ca	Mg	S	Cu	Mn	Zn	B	Fe
NPK	Mean	3967	2.52	0.28	1.03	1.02	0.34	0.73	5.53	138.0	17.8	2.78	42.0
	Min	1419	1.77	0.12	0.53	0.61	0.19	0.04	2.91	93.8	10.7	0.62	25.4
	Max	5769	3.37	0.39	1.70	1.61	0.52	1.20	8.84	223.8	30.6	5.85	73.7
	CV (%)	33	15	24	33	28	27	49	35	29	29	43	27
NPKB	Mean	2386	2.68	0.24	1.13	1.11	0.34	0.76	4.33	156.0	16.1	2.24	38.3
	Min	599	2.08	0.15	0.51	0.58	0.17	0.15	2.24	98.2	0.6	0.91	25.5
	Max	3986	3.59	0.33	1.74	1.73	0.52	1.29	8.19	237.2	24.5	3.95	83.9
	CV (%)	16	20	34	27	31	41	23	40	38	39		
NPKS	Mean	3776	2.69	0.27	1.18	1.23	0.36	0.71	5.15	145.4	17.4	2.60	51.4
	Min	1673	2.19	0.10	0.72	0.78	0.22	0.01	2.84	80.9	7.8	0.80	25.4
	Max	7450	3.49	0.37	1.94	2.04	0.51	1.18	8.82	203.5	26.4	4.54	148.6
	CV (%)	38	15	23	30	25	22	42	37	23	28	35	59
NPKZn	Mean	5097	2.62	0.24	1.24	1.15	0.34	0.84	5.54	142.7	23.5	2.20	51.9
	Min	3122	2.07	0.14	0.82	0.69	0.20	0.33	2.83	88.3	17.1	0.47	35.4
	Max	6535	3.21	0.39	1.79	1.70	0.53	1.28	10.04	202.7	40.8	3.49	73.5
	CV (%)	22	11	29	24	24	27	39	35	20	28	45	22
NPKSZnB	Mean	4093	2.52	0.23	1.16	1.19	0.39	0.50	4.79	158.8	19.9	2.84	44.3
	Min	2004	2.09	0.15	0.43	0.58	0.17	0.01	2.83	100.4	11.5	1.83	25.4
	Max	6887	2.89	0.32	1.97	2.16	0.78	1.16	8.21	241.0	36.9	4.39	85.9
	CV (%)	43	11	24	33	33	39	72	32	21	34	22	35

### Ear leaf nutrient concentration

Ranges of nutrient ear leaf concentrations across the treatments is shown in Tables 2 and 3. All treatments with N application resulted in higher N ear leaf concentration compared to the Control and PK treatments. Phosphorus (P) concentration ranged from 0.04% in Control treatment to 0.39% in NPK and NPKZn treatments. Higher average P ear leaf concentrations were observed for the NP, PK and all NPK treatments, and were significantly different from that of the Control (0.16%) and the NK (0.20%). For K, there was no significant differences between the various treatments, though the NK and NPK + treatments had slightly higher ear leaf K concentrations. The omission of K did not reflect in lower average ear leaf concentration. The highest average K ear leaf concentration was observed for the NK treatment. There was no indication that K application in itself led to higher K concentrations in the ear leaf. Average ear leaf concentration of Ca was below 1.23% across the treatments. However, Ca concentration was higher in NPK and NPK + treatments. Magnesium (Mg) concentration ranged from 0.01 to 1.46%. There was little difference in the average Mg and Ca concentrations for the various treatments, only that the Control had a lower concentration for the elements. Sulphur concentration ranged from 0.01 to 1.29% across all treatments. With the exception of NPKSZnB treatments (0.05%), the S ear leaf concentration for all the NPK related treatments was slightly elevated (ranging from 0.71 to 0.84% on average) compared to the Control, NP, NK and PK treatments, which seems to suggest some relationship to the yield level.

There was no clear link between the micronutrients in the ear leaf to any of the treatments. Highest concentration of Cu was 11.25 mg kg^−1^ for the Control treatment and the lowest concentration of 2.24 mg kg^−1^ was found for the NPKB and PK treatments. Manganese (Mn) concentration was generally higher in plots where N was applied. There was an average of 35.00% increase of Mn ear leaf concentration across N treatments compared to when N was omitted and for the Control. Among the NPK and NPK + treatments the mean Zinc (Zn) concentration was highest for the NPKZn (23.50 mg kg^−1^) and the NPKSZnB (19.9 mg kg^−1^) treatments, but not different from the NP and NK treatments (~ 24.0 mg kg^−1^). This suggest a possible effect of the Zn application, whereby the dilution of the Zn concentration in the higher yielding treatments was compensated by the Zn application under the NPKZn and NPKSZnB treatments. Boron (B) concentration in the ear leaves ranged from 0.33 to 5.85 mg kg^−1^. Surprisingly lower B concentrations were found for the NPKB and NPKZn treatments. Concentration of Fe was averagely high across the treatments, with highest means recorded with NPKS and NPKZn treatments.

### DRIS indices

The DRIS ratios were selected based on higher variance ratio between the low and high yielding sub-populations. The variance ratios of all the selected dual ratios of the low against the high yielding sub-populations were ≥ 1, which indicate relative higher variance of the low yielding sub-population. The variance ratios of N/K (153.72) and B/Zn (117.97) (Table 4) were the highest among the ratios.

**Table 4:** Some selected maize DRIS norms, dual ratios and variance used in the nutrient diagnosis.

Form of expression	Low yielding sub-population			High yielding sub-population			Variance ratio
	Norms	CV (%)	σ^2^_low_	Norms	CV (%)	σ^2^_high_	σ^2^_low_/ ^2^_high_
N/Mg	8.889	158.3	197.968	8.530	33.3	8.051	24.59
N/K	3.615	319.1	133.064	4.460	37.8	0.866	153.72
N/Ca	2.869	118.0	11.452	2.420	20.9	0.257	44.64
N/P	12.126	50.5	37.532	14.199	29.8	11.142	3.37
N/Zn	0.150	219.0	0.155	0.168	37.4	0.003	50.91
Mg/K	0.403	72.1	0.084	0.627	53.7	0.031	2.72
Mg/Ca	0.384	42.2	0.026	0.481	33.4	0.011	2.49
P/Mg	0.787	126.6	0.993	0.821	39.9	0.107	9.26
S/Mg	2.358	161.9	14.569	2.497	51.3	1.643	8.87
Cu/Mg	0.210	256.4	0.289	0.177	45.7	0.007	44.37
Mn/Mg	5.345	208.5	124.179	4.730	34.6	2.681	46.32
Fe/Mg	1.524	153.2	5.456	1.571	54.0	0.718	7.59
B/Mg	0.111	242.7	0.072	0.718	47.9	0.001	60.80
K/Ca	1.189	42.7	0.257	1.609	36.1	0.160	1.61
P/K	0.307	262.1	0.648	0.332	39.8	0.009	75.97
S/K	0.705	321.5	9.049	0.936	58.3	0.169	53.58
Fe/K	0.606	312.5	3.592	0.472	72.2	0.116	30.96
P/Ca	0.258	93.7	0.059	0.337	39.3	0.009	6.76
S/Ca	0.767	123.1	0.893	0.793	51.9	0.141	6.35
Fe/Ca	0.481	115.7	0.309	0.517	36.9	0.026	11.90
B/Ca	0.026	180.0	0.004	0.041	50.3	0.000	38.42
S/P	3.377	107.2	13.092	3.383	57.1	3.563	3.67
Cu/P	0.250	61.9	0.026	0.265	43.2	0.009	2.75
Mn/P	6.976	47.1	10.814	6.431	42.8	7.582	1.43
P/Zn	0.011	277.2	0.003	0.019	43.0	0.000	72.02
Fe/P	2.074	51.6	1.145	2.122	42.9	0.754	1.52
B/P	0.142	53.1	0.006	0.094	48.1	0.002	2.79
S/Zn	0.045	158.5	0.005	0.046	54.6	0.001	9.48
Fe/Zn	2.811	164.7	2.930	2.811	71.3	4.023	5.79
B/Zn	0.245	307.9	0.571	0.313	55.6	0.005	117.97
B/Fe	0.035	41.6	0.001	0.054	54.4	0.001	1.12

Percentage of plots with negative DRIS indices for the nutrient treatments (Table 5) indicates that PK, NPKS and NPKZn treatments have highest percentage of plots with negative DRIS nutrient indices. Among the nutrients, B had the lowest percentage of plots with negative index values across the nutrient treatments, while Fe had the highest number of negative index plots. Among the nutrient omission treatments, the PK treatment showed the highest frequency of plots with negative N index which reflects the impact of the N limitation on the DRIS index value. The NK, NP and NPK treatments showed lower percentage (33.30 and 40.00% respectively) of plots with negative N DRIS index indicating that N was imbalanced due to higher concentrations of other nutrients. The percentage of plots with negative N DRIS index for the NPK and NPK + treatments ranged from 40.00 to 64.30% apart from the NPKS treatment which had 84.60% of plots with a negative N DRIS index value. The slightly higher percentage for the NPKZn treatment indicates a possible dilution of the N concentration on the ear leaf because of the higher yield.

**Table 5:** Percentage of fields with negative DRIS indices for various nutrient treatments.

Treatment	Nutrient negative index (%) plots										
	N	P	K	Mg	Ca	S	Cu	Mn	Zn	Fe	B
Control	58.3	58.3	66.7	100.0	50.0	50.0	41.7	66.7	83.3	83.3	16.7
NK	33.3	75.0	91.7	66.7	25.0	66.7	58.3	66.7	58.3	66.7	33.3
NP	40.0	73.3	33.3	73.3	33.3	53.3	46.7	60.0	80.0	66.7	33.3
PK	78.6	78.6	64.3	85.7	85.7	71.4	71.4	85.7	50.0	100.0	14.3
NPK	40.0	80.0	53.3	66.7	40.0	53.3	60.0	66.7	46.7	73.3	33.3
NPKB	46.7	66.7	46.7	60.0	60.0	53.3	80.0	60.0	53.3	66.7	40.0
NPKS	84.6	76.9	61.5	76.9	61.5	61.5	61.5	76.9	53.8	92.3	46.2
NPKZn	64.3	64.3	71.4	78.6	71.4	57.1	71.4	78.6	92.9	85.7	42.9
NPKSZnB	53.8	84.6	76.9	76.9	46.2	46.2	61.5	53.8	61.5	92.3	30.8

The percentage of negative P DRIS index value plots was lowest for the ‘Control’ treatment (viz. 58.30%) and for the other treatments the percentage did not differ much, with the percentage ranging between 64.30 to 84.60%, indicating that for all treatments the relative P concentration is lower than for the reference population. There was no marked decrease in percentage of the plots with negative P DRIS Index value for the NP and PK treatments or a marked increase in percentage for the NK treatment (P-omission treatment).

Omission of K (NP treatment) resulted in a reduced percentage (33.3%) of negative K index plots, contrary to what would be expected and the NK treatment showed an unexpected high percentage of plots with negative K DRIS index values. Otherwise, the percentage of negative plots varied between 46.75 and 76.90% indicating that the relative K concentration was generally lower than for the norms where K was involved. The NPKS increased number of plots with negative Fe index by 19.00% and that of B by 13.00% compared to NPK. Ca index however, becomes increasing negative in plots where Zn was applied to or N was omitted from NPK. Both the PK and NPKZn treatments resulted to relatively higher percentages of plots with negative DRIS index scores for most of the nutrients. For the NPKZn, this was also true for most nutrients except for the N and P DRIS indices and to lesser extent for the S DRIS index score. The generally lower percentage of plots with negative B DRIS index values, across the various treatments, indicates that the relative B ear leaf concentrations of the low sub-populations was not much different, which probably indicates that the B concentration was low across the board. On the other hand, the percentage of negative plots with negative Fe DRIS index values was relatively high, irrespective of the treatment, indicating that the reference population of plots with high yields have relatively lower Fe concentration in the ear leaves. The same applies to Mg, where we find high percentages of plots with negative DRIS scores across the treatments, indicating that the reference population has a relatively low Mg ear leaf concentration, indicating a possible nutrient limitation.

Table 6 shows the ranking of the nutrient limitations based on the DRIS index value for each treatment. For the interpretation one has to take account of the treatment and the nutrients. N ranked high (2nd) in the order of nutrient limitations for the PK treatment, while it ranked lower for other treatments where N was applied, indicating N was highly limiting. For P and K, the pattern was less clear and also not ranking highly in terms of nutrient limitations for the respective nutrient omission treatments. Sulphur (S) ranked high in the order of nutrient limitations for the NK, NP and PK treatments as well as for the NPK and NPKSZnB treatments. It ranked low for the NPKS and the NPKZn. This suggest that S was clearly a limiting nutrient, with the low ranking for the NPKZn explained by the possible positive effect of Zn application on the availability of S. Zinc (Zn) ranked high in the order of nutrient limitations for the ‘Control’, NK and NP treatments, but did not seem to be specifically prominent for the other high yielding treatments. Boron ranked lowest based on its DRIS index value for seven of the nine treatments and ranked highest for the NPKZn treatment. Note that the NPKZn treatment generated the highest yields and the corresponding plots will therefore constitute a large part of the reference population. The negative interaction with B thus explains the generally low percentage of plots with negative B DRIS index scores and consequently low ranking in the order nutrient limitation for the remaining treatments other than NPKZn. Therefore, the low ranking in order of nutrient limitation in this case does not necessarily signify that B is not a limiting nutrient.

**Table 6:** Order of nutrient limitation for the nutrient treatments.

Treatment	Order of nutrient limitation
Control	B > P > S > Cu > Ca > N > Fe > Mn > K > Zn > Mg
NK	B > Cu > N > Ca > Mn > Fe > P > Mg > K > S > Zn
NP	B > Cu > K > Ca > N > Mn > P > Fe > Zn > Mg > S
PK	B > Ca > P > Zn > Cu > Mn > K > S > Fe > N > Mg
NPK	B > Zn > Ca > K > N > Cu > Mg > P > Mn > Fe > S
NPKB	B > Mn > S > N > Zn > K > Fe > P > Ca > Mg > Cu
NPKS	B > P > S > Zn > N > K > Mn > Fe > Ca > Mg > Cu
NPKZn	S > Ca > Cu > P > N > Mg > Zn > Fe > K > Mn > B
NPKSZnB	K > Zn > N > B > Mn > P > Cu > Fe > S > Mg > Ca

With regards to the nutrients that are not included in any of the treatments, Mg ranked high in the order of nutrient limitations for all the treatments (often ranking first, second or third). This is also consistent with the relatively high percentage of plots with negative Mg DRIS index values. Therefore, among this group Mg is considered the most important yield limiting nutrient. Secondly, Fe ranked relatively high, ranking second to sixth depending on the treatment. Also, in this case it is consistent with the generally high score of plots with negative Fe DRIS index values across the treatments. No particular evidence of Mn limitation was observed in any of the treatments. Noteworthy is the high ranking in order of nutrient limitation for the NPK (3^rd^) and the NPKZn treatment (2^nd^), indicating possible interaction with Zn application. A similar observation was made for Ca and Cu, in that there was no particular evidence that the nutrients might be limiting, but that for particular treatments the high ranking is noteworthy. In this case the NPKS, NPKB and the NPKSZnB treatments show high ranking of Ca and Cu (Table 6) suggesting an interaction with S and B on the availability of these nutrients.

### Relationship between ear leaf nutrient concentrations and DRIS indices

**Figure 1:**
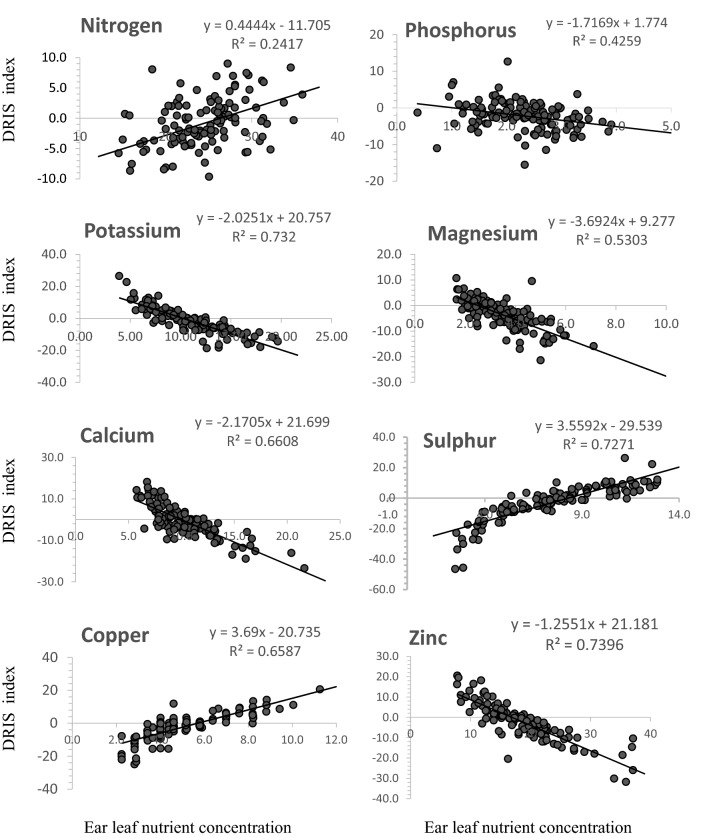
Relationship between ear leaf nutrient concentrations and corresponding nutrient DRIS index.

Figure 1 shows varied strengths of relationship between ear leaf nutrient concentration and corresponding DRIS index values from weak to strong. The Figure shows that ear leaf nitrogen concentration is a poor indicator of N index in DRIS with a low R^2^ value (0.24). The figure also shows a strong proportionate increase in DRIS index values of S and Cu at corresponding higher ear leaf nutrient concentrations. The DRIS index values of K, S and Zn were the most strongly explained by ear leaf concentration among the analyzed nutrients (R^2^ = 0.73). Higher ear leaf nutrient levels of K, Mg, Ca, and Zn correspondingly showed strong influence for consistent decrease (negativity) in DRIS indices.

### GGE biplot analysis

**Figure 2:**
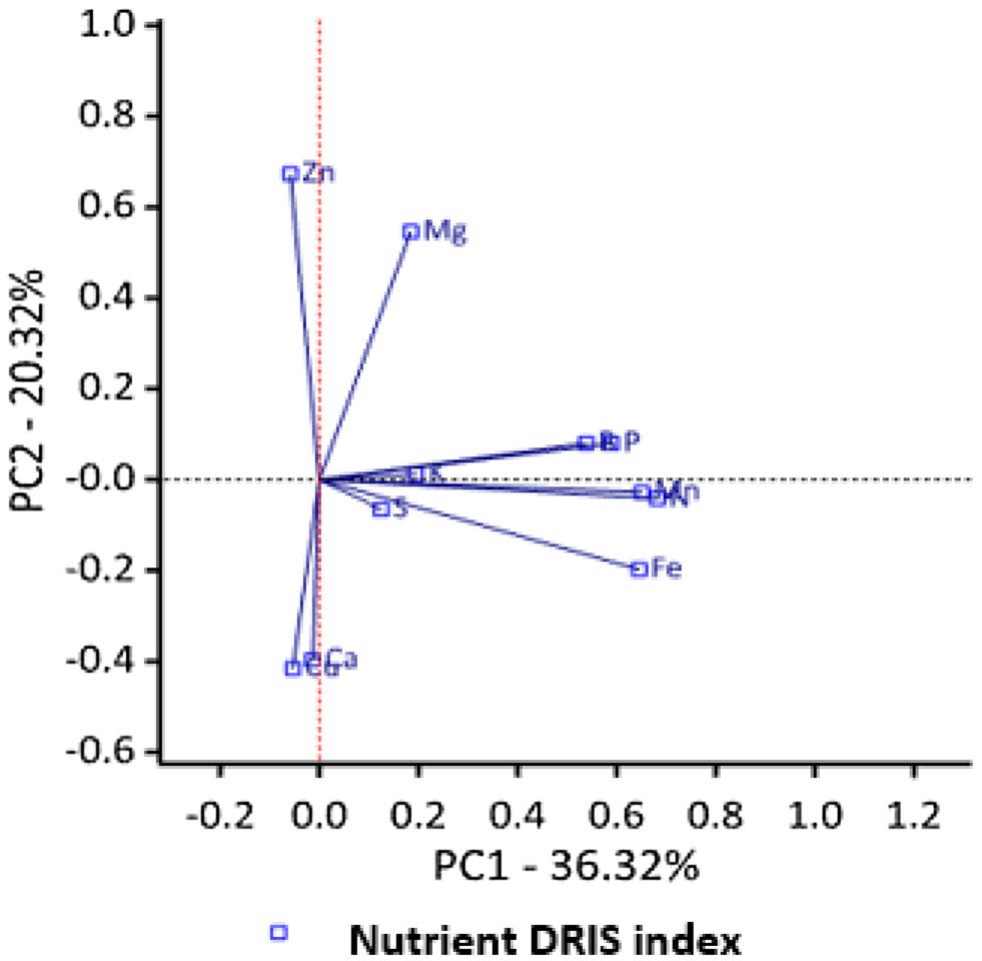
Relationship among nutrients DRIS indices and maize grain yield.

Figure 2 shows relationship among the nutrient DRIS indices on a biplot. PC1 explained 36.32% of the total variation in the data. The PC1 was strongly influenced by the positive correlations with the DRIS indices Mn, N, Fe, P and B. Additional 20.32% of the variance was explained by PC2, and was positively correlated with index of Zn with negative correspondence with Cu index. Indices of P, B, Mn, N, B, K, and S were positively correlated with each other; indicating a synergistic relationship. Indices of Zn and Mg were positively correlated with each other and negatively with Cu and Ca, indicating a possible antagonistic association between them.

**Figure 3:**
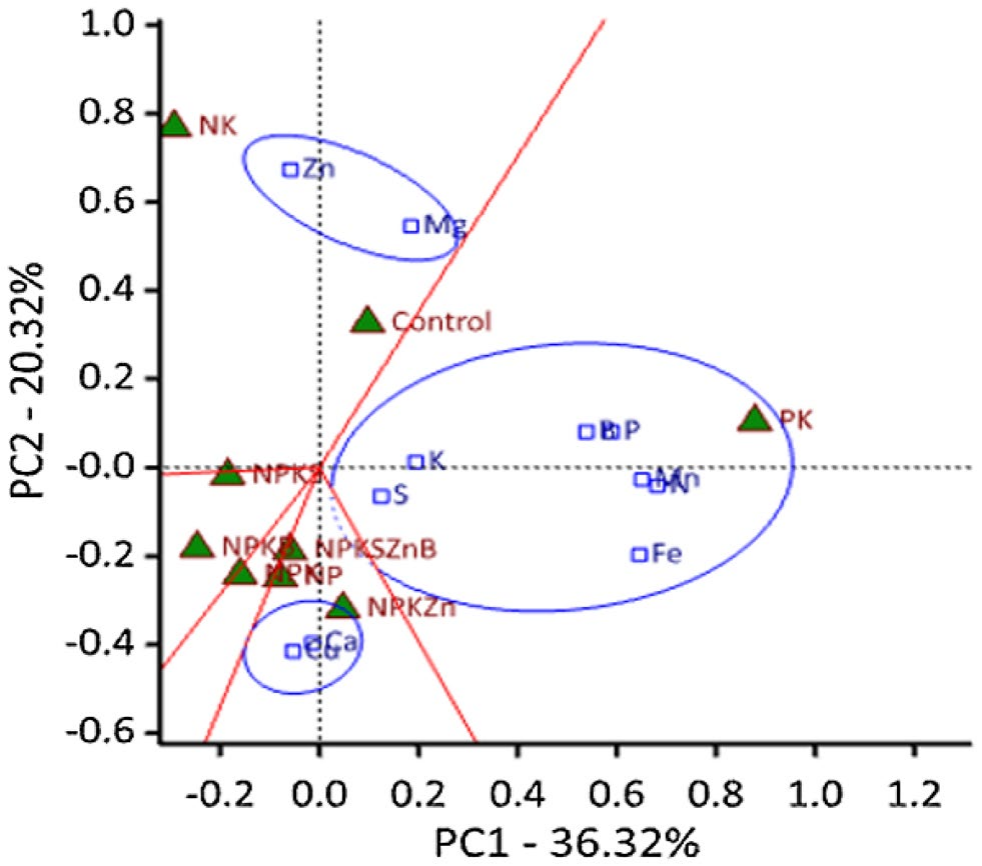
Which-Won-Where biplot showing the effect of nutrient treatments on DRIS indices and yield.

The ‘Which-Won-Where’ view of the GGE biplot (Fig. 3) show the degree of influence of nutrient treatments on the nutrients DRIS indices. The general rule in GGE is that nutrient indices that share the same sector with a particular nutrient treatment are the most associated with that treatment. The indices of Zn and Mg formed a cluster and share the same sector with Control and NK treatments. The largest cluster was formed by the indices of K, S, P, Mn, Fe, B and N, and found in the sector of PK treatment. This sector is bordered closely by Control, indicating that these two treatments have similar DRIS index values for most of the nutrients that are within that cluster. In the diagnosis, Cu and Ca indices became more consistently important yield limiting under NPKZn and NPKSZnB. The DRIS nutrient indices associated with NPK, NPKS and NPKB did not show any consistent pattern of occurrence.

## Discussion

Maize yield was significantly affected by the nutrient treatments. The results reflected the responsiveness of maize to the applied nutrients compared to when no nutrient was applied. As reported by Shehu et al.^5^, maize yield can reach > 5 tons per hectare in northern Nigeria when nutrient deficiencies are properly addressed. Application of NPKZn gave the highest yield. This indicates relative response to Zn in the study area. The result confirmed the findings of Garba et al.^40^, that Zn application increased maize yield in some parts of Nigeria. The low CV of yield under NPKZn treatment in this study also confirmed the stability of maize yield response to Zn in the study area. This may be due to the improvement of fertilization uptake caused by Zn application^41^. Yield decrease due to B application observed in this study has also been previously reported by Shehu et al.^5^. Results of the soil analysis also confirmed B deficiency in the soils in this study, however, since there is dearth information about B application rates, it is possible that the B application rate used in this study was higher than required, and might have resulted to B toxicity, or lower than required for significant B response. In this sense it can be argued that the high amount of soil Zn might have countered B expression as similarly reported by Rehman et al.^42^.

Occurrence of negative DRIS index of N was higher in the treatments where no nitrogen was applied (Control and PK) or when other nutrients were applied with NPK (especially NPKS and NPKZn). A significant increase in ear leaf concentration of N beyond the critical level of 2.60% when N was applied also indicated that maize responded to N treatments. The relationship between N DRIS index and N ear leaf concentration discovered in this study indicated a proportionate increase in N balance index at higher ear leaf concentrations. But since this relationship is weak, emphasis could not be made concerning the negativity of the N balance index in some plots where N was applied. In a similar study, Reis Júnior et al.^43^, Silveira et al.^44^, Nachtigall and Dechen^45^ observed very low correlation coefficients for N and S between their ear leaf concentrations and respective DRIS balance indices, indicating that other important factors play role in the nutrient balance. The current study was on-farm that cut across wide range of cropping potentials. This could have possibly caused a large variation in the ear leaf content of nutrients due to different previous nutrient management which affected the N balance. In progressive diagnosis, Sumner^46^ discovered that negative N index was not an indication of deficiency, but an imbalance caused by continuous application of P, S and K. Similarly, the high percentage of negative N index plots of NPKS and NPKZn treatments (compared to the NPK) might not directly be related to N deficiency, rather an imbalance as a result of S and Zn additions. This can be attributed to the very low variance between the dual nutrient ratios (N/S and Zn/N) of the sub-populations^47^. It can therefore be concluded that N diagnosis using these treatments is likely ineffective in this study. The K ear leaf contents for NK and NPKZn were within the sufficiency range (1.20–1.70%) for the region reported by Reuter et al.^48^, this thus indicates some level of K response. However, K application under PK and NPK, showed a significantly lower K leaf concentration below the critical limit. Therefore, it can be concluded that K response is likely a site-specific scenario. The strong inverse relationship between K ear leaf content with DRIS index indicates that K application negatively increase K imbalance in many situations. Further, the table for order of nutrient limitation indicated that K was limiting in both scenarios of omission and application. As indicated by Kihara et al.^49^ response to K may be very patchy. Though the lower yield of NP observed indicates the significance of K, the large variation in the results (CV is 65%) further confirmed variable response to K in the area. Shehu et al.^5^ and Nziguheba et al.^10^ both indicated that K is not a limiting nutrient for maize production in some soils within the study area. Inconsistent response to K could be attributed to the large deposit of K-feldspars as the dominant mineral^28^ in rocks of the basement complex and dust deposition of K during the harmattan^10^ which resulted in inherent high K concentrations in the soil, but these may have been depleted depending on the type of land use and land use history and caused variable response.

The ear leaf concentration of P was below the critical limit (0.27%) established by Reuter et al.^48^, apart from those of NPK and NPKS treatments (0.28% and 0.27% respectively). Effect of P application did not directly reflect in the P balance index except for the NPKS where P was the second least important limiting nutrient. The higher ear leaf content and lower limitation order of P under the NPKS is not surprising as P and S interact in synergy to influence uptake of each other. Studies in Nigeria by Ogunsola and Adetunji^50^ recommended S application for enhanced P uptake by maize. The high recurrence of negative DRIS P balance index and increased order of P limitation for NPK and NPKB treatments is more difficult to explain, especially as P uptake is reported to be positively influenced by B application^51^. But since B is not an important limiting nutrient in most of the diagnosis results, it is likely that rate of B applied in this study might have affected the P balance. The strong positive correlation between P and B indices in this study further confirms the effect of B on P. Also, most of selected dual nutrient norms that involved P for the high yielding sub-population (which were used for the diagnosis) were not much higher than their corresponding low yielding sup-population. This means that the DRIS index calculated for P involved some samples from high yielding but unbalance sub-population, and this must have affected the P DRIS index. Though the norms involving P in this study are in most cases higher than those obtained by others like Serra et al.^52^ and Anabela et al.^53^ in other continents, yet the ear leaf P contents were below the sufficiency limit, this therefore suggest that nutrient norms cannot be universally adopted.

Range of Ca concentration in the ear leaves was small as indicated by a low CV (< 25%) for most of the treatments. The average concentration of Ca was obviously above the critical concentration levels of 0.21% and 0.30% established by Reuter et al.^48^ and Nziguheba et al.^10^ respectively. Although, Ca was not a treatment in this study, some amount of Ca (~ 15%) is contained in TSP fertilizer that was used as source of P in this study, might be the reason for the high Ca ear leaf concentrations, especially where P was applied. Negative DRIS Ca index is also confirmed to be associated more with the NPK treatments as shown in the Which-Won-Where plot. The percentage fields with negative Ca index was also lowest in the NK confirming the earlier suggestion. In the WBE experiment, Nziguheba et al.^10^ also reported negative Ca index, even though ear leaf concentration of Ca was above the critical limit, and they concluded that the negativity indicated Ca was imbalanced not deficient. Therefore, we conclude also that the negative Ca index observed in this study translates to imbalance. This is further confirmed by the high level of Ca content in the soils observed in this study. Adequate amount of Ca was reported by Agbenin^54^, in most soils in West African savanna except in cases of long-term continuous cultivation and without Ca application. Negative S index was more associated with PK treatment and less with NPKS and other treatments. Contrary to the conclusion made by Nziguheba et al.^10^ that pronounced imbalances of Mg and Ca hide S effect, our results revealed that S is next most critical nutrient after deficiencies of N, P and K were addressed.

The average B ear leaf concentration for all the treatments was within the sufficiency range of 2–5 mg kg^−1^ established for maize by Kelling^55^. The B treatments resulted in relatively higher B DRIS index. However, it did not reflect in the B leaf concentration, which was relatively lower for the NPKB treatment. The results were similar to that of Aref^51^, where the Control and B treatments were not significantly different for B ear leaf concentration. The occurrence of negative index of B across the treatments was however lower compared to those of the other nutrients. Boron (B) was also shown to be least important limiting nutrient except under NPKZn. Though previous studies^56^ have showed widespread deficiency of B in the soils within this study area, but considering the very narrow range (0.3–1 mg kg^−1^) between deficiency and excess levels for soil B, it could be assumed that the B application rate (5 kg ha^−1^) used in this study might have been high enough to have caused B toxicity, and subsequently, the low yield of the B treated plots. The Zn ear leaf concentrations were higher than those reported by Nziguheba et al.^10^ in the same region. Our findings seem to suggest an effect of Zn application on the ear leaf content, with the NPKZn and the NPKSZnB treatments showing slightly elevated Zn ear leaf concentration among the NPK treatments. The Zn ear leaf concentration for the NK and NP treatments were relatively high (though not significantly different from the other treatments) which also seems to be reflected in the higher Zn DRIS index values (− 8.4 and − 3.1 respectively—not shown). The pronounced limitations by Ca and Cu seem to have masked the effect of Zn in many of the treatments. Other analyzed micronutrients (Cu, Mn and Fe) in this study were mostly above their critical concentration levels with all treatments except when N was not applied, or when B was applied.

## Conclusions

The nutrient treatments used in this study significantly affected the ear leaf nutrient concentration, DRIS indices, and maize yield. Omission of any of the major macro nutrient (N, P and K) resulted in lower yield than when they were applied. Ear leaf concentrations of most nutrients correlated well with their corresponding DRIS indices. The DRIS method identified nutrient imbalance for maize production in the maize belt of Nigeria. The diagnosis indicated consistent imbalances of unused secondary macro and micronutrients (Mg, Cu and Ca) in most NPK treatments; revealing their relative importance in maize nutrient management in the area. The Which-Won-Where showed that the frequent occurrence of negative imbalances of nutrients associated with PK treatment is systematic rather than random. None of the diagnosed nutrients is within the balance range of − 1 to + 1 even under the highest yield treatment (NPKZn); indicating that yield can be further increased when nutrient imbalances are further addressed. DRIS diagnosis is complementary to soil analysis in diagnosis of nutrient limitations and appropriate method to making recommendations on balanced crop nutrition. Balanced nutrition of maize in the maize belt of Nigeria should target the application of varying rates of N, P, K, Mg, S and Zn, depending on the soil condition. Because of complexities of nutrient interactions during uptake, it is hardly possible to realize a balanced nutrition. However, differentiating the application of antagonistic nutrients (especially the divalent cations) into foliar or soil methods is recommended for a more balanced maize nutrition.
